# Supplementary material for: Chiral emergence in multistep hierarchical assembly of achiral conjugated polymers
Source: Nat Commun. 2022 May 18;13:2738. doi: 10.1038/s41467-022-30420-6 (PMC9117306; doi:10.1038/s41467-022-30420-6)
Supplement: Supplementary file 1 — Supplementary Information [file 41467_2022_30420_MOESM1_ESM.pdf]

Supplementary Information

Kyung Sun Park[1], Zhengyuan Xue[1], Bijal Patel[1], Hyosung An[2], Justin J. Kwok[2], Prapti Pkafle[1], Qian Chen[2], Diwakar Shukla[1] and Ying Diao[1,2,3,4,5]*

1Department of Chemical and Biomolecular Engineering, University of Illinois at Urbana-Champaign, 600 S. Mathews Ave., Urbana, IL 61801, USA.

2Department of Materials Science and Engineering, University of Illinois at Urbana-Champaign, 1304 W. Green St., Urbana, IL 61801, USA.

3Beckman Institute, Molecular Science and Engineering, University of Illinois at Urbana-Champaign, 405 N. Mathews Ave., Urbana, IL 61801, USA.

4Department of Chemistry, University of Illinois at Urbana-Champaign, 505 S. Mathews Ave., Urbana, IL 61801, USA.

5Materials Research Laboratory, The Grainger College of Engineering, University of Illinois at Urbana-Champaign, 104 S. Goodwin Ave., Urbana, IL 61801, USA.

*Corresponding author. Email: yingdiao@illinois.edu

Supplementary Methods

Supplementary MD simulation details

MD simulation on long PII-2T chain (30mer) was performed to investigate the conformation of actual polymer chains in chloroform solvent. Moreover, simulations on PII-2T oligomers (hexamer) with removal of alkyl chains were performed to investigate the behavior of PII-2T oligomer in chloroform and explore the source of assembly and chirality emergence. Lastly, umbrella sampling simulations were performed to study the relationship of interaction between backbones and position of the aromatic rings. Simulation details are summarized in the table below:

Supplementary Table 1. MD simulation parameters.

System	Polymer length	Polymer position	$CHCl_3$	Simulation Box (Å)	Simulation Time (ns)
30-unit PII-2T chain	30	Fixed	29233	104.0*99.0*448.6	262
2 hexamer with removal of alkyl chains	6	Dynamic	4534	90.9*114.5*75.0	400

<u>Initial Setup of 30-unit polymer simulations.</u> The starting structures of PII2T monomers were drawn using PubChem Sketcher[1] and optimized by the built-in structure optimization function of Avogadro[2]. The monomer topology file was generated by Antechamber and using the general AMBER force field (GAFF)[3]. The 30mer chain structure model was generated by repeating the monomer structure for 18 times in Antechamber. Periodic simulation box of 448.6 Å in length with the central ax alongside with the 30mer was generated using tLEaP from AmberTools. The system was then solvated with 29233 $CHCl_3$ molecules, and topology file was generated using tLEaP[3].

<u>Initial Setup of cut side-chain hexamer simulations.</u> The starting structures of PII2T monomer

with removal of alkyl chains was generated using PyMol[4]. The hexamer chain structure model was generated by repeating the monomer structure for 6 times in Antechamber[3]. System with periodic simulation box of the size 90.9*114.5*75.0 Å and 2 hexamer putting parallelly with 20 Å apart at the center was generated using Packmol[5]. The system was then solvated with 4534 $CHCl_3$ molecules in tLEaP[3].

All the simulations were set up using the AMBERTools18 and performed with AMBER18 software using the general AMBER force filed (GAFF)[3]. All the partial charges were derived using the AM1/BCC method. The same partial charges from monomer PII-2T were used for each unit of the PII-2T polymer chain. Previous studies have shown that the GAFF can accurately reveal behaviors of PII-2T in chloroform solvent[6]. Both the systems was minimized using steepest descent method for 9200 steps and then slowly heated up to 300 K in 20 ps before simulation.

All the simulations were performed in NPT ensemble (1 atm, 300K) with periodic boundary conditions. Particle-mesh Ewald method was used to treat the electrostatic interactions with a 10Å cutoff distance[7]. The SHAKE algorithm was applied to constrain the length of covalent bonds involved hydrogen atoms to their equilibrium values[8]. The integration step was 2 fs. Berendsen thermos-barostat with a damping time constant of 2 ps was used to control the temperature and pressure of the ensembles[9].

Umbrella Sampling Simulation and Data Analysis. All umbrella sampling simulations were run in Amber18[3], with same basic setting as previous simulations. Amber's harmonic restraints were used for restricting the distance or dihedral angle. In the umbrella sampling using COM distance as a collective variable, we used umbrella windows spaced 0.5 Å apart, and a force constant of 20 kcals/mol Å. The umbrella windows were covering the distance from 1 to 13 Å, and the simulation was run on each window for 10 ns. During the following data analysis, since

the distance could not go below 3 Å, that part of the data was cut. In umbrella sampling using dihedral angle as a collective variable, we used umbrella windows spaced 3 degrees apart, and a force constant of 200 kcal/mol rad^2. The umbrella windows were covering dihedral angles from -180 to 180 degrees, and the simulation was run on each window for 2 ns.

Weighted Histogram Analysis Method (WHAM) were used to analyze the data from umbrella sampling simulation and generate the PMF[10, 11]. The PMF were generated with assumed temperature at 300 K and no padding. And statistical error was estimated using Monte Carlo bootstrap error analysis, with 10 fake data sets generated using random seed of 5.

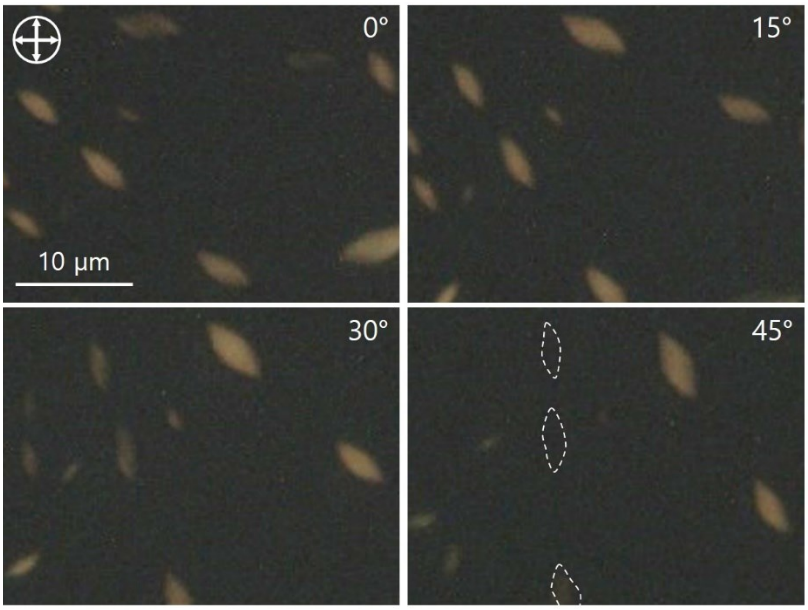

Supplementary Figure 1. Homogeneous tactoids observed at 50 mg/ml PII-2T solution. The major axis rotated clockwise with respect to a polarizer by 0◦, 15◦, 30◦, and 45◦. Homogenous tactoids show a uniform change in brightness when the tactoid is rotated from 0◦ to 45◦ relative to either of the polarizers, and are uniformly dark at 45◦.

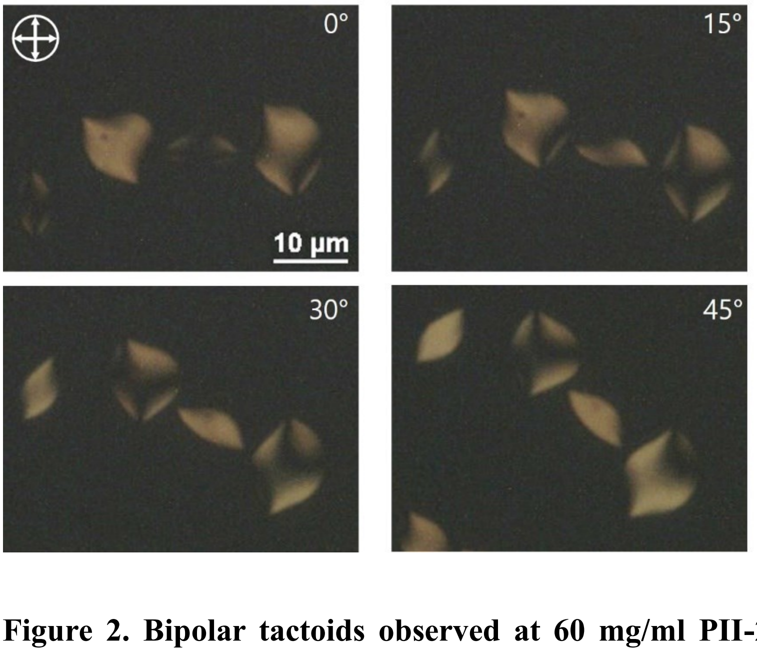

Supplementary Figure 2. Bipolar tactoids observed at 60 mg/ml PII-2T solution. The major axis rotated clockwise with respect to a polarizer by 0◦, 15◦, 30◦, and 45◦. Bipolar tactoids show four dark brushes crossing at the center when either of the crossed polarizers is aligned with the major axis of the tactoid. By rotating the tactoid, the dark cross splits into two dark curved brushes that move away from each other.

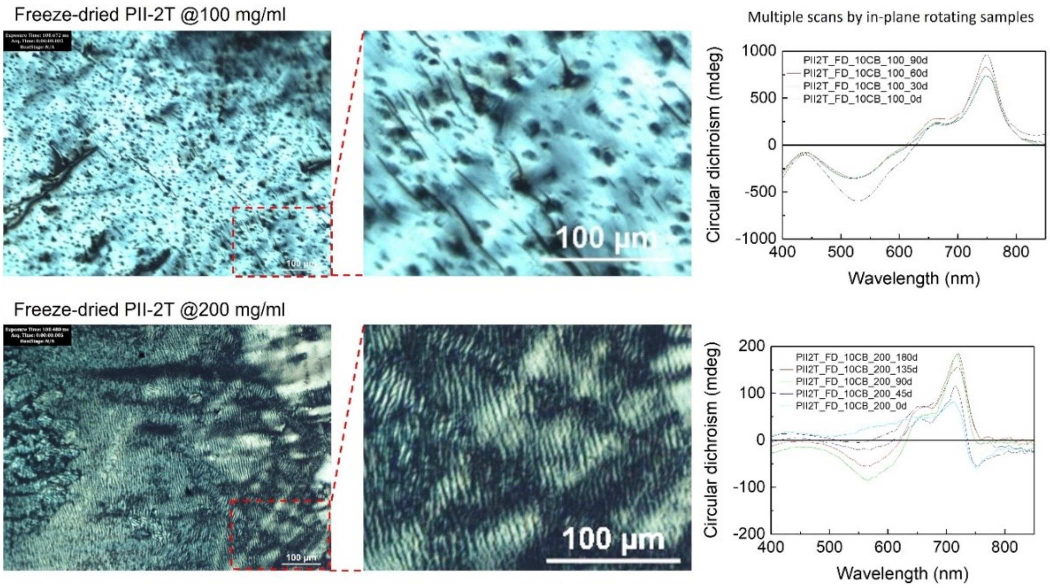

Supplementary Figure 3. Cross-polarized optical microscope images and corresponding CD spectra of the freeze-dried PII-2T prepared at 100 and 200 mg/ml, respectively. CD measurements were carried out with the sample rotated multiple in-plane angles to rule out the linear dichroism and birefringence. The CD spectra show the chirality is preserved after removing the solvent during the freeze drying process.

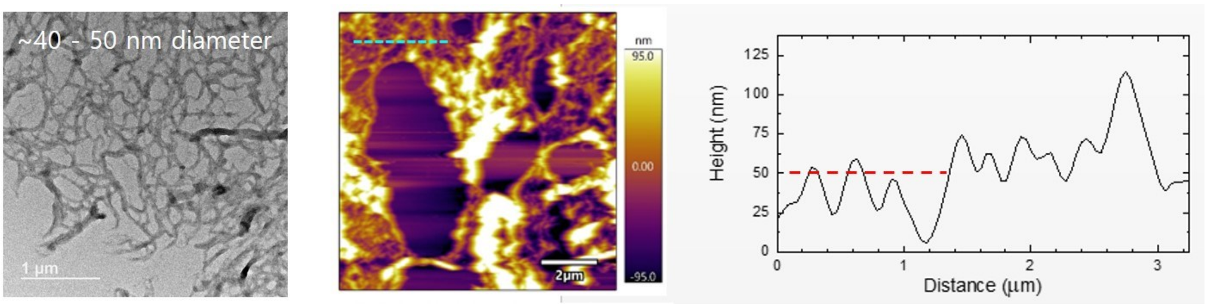

Supplementary Figure 4. TEM and AFM images of 10 mg/ml PII-2T freeze drying samples. The cross section of fibers is almost circular which is estimated by measuring the height of individually dispersed fibers.

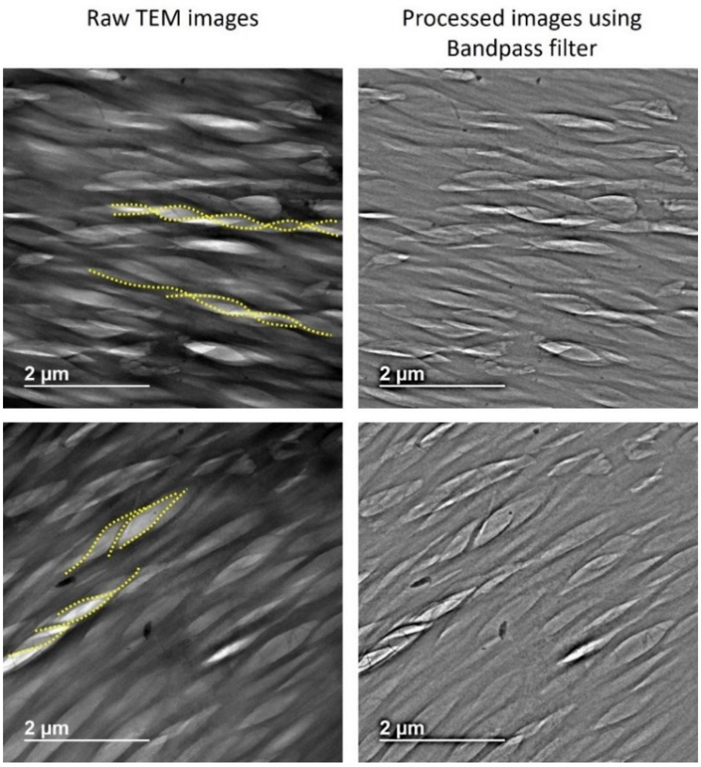

Supplementary Figure 5. TEM images of freeze-dried PII-2T mesophase at 100 mg/ml. The yellow dotted lines in images exhibits the twisted structures. Further imaging analysis was performed using ImageJ.

Supplementary Table 2. Molecular stacking distance [Å] (top) and the full width at half maximum (FWHM) [Å^{-1}] (bottom) obtained from GIWAXS measurements.

		Isotropic phase	*Nematic tactoids*	*Twist-bent mesophase I*	*Twist-bent mesophase II*
π-π stacking	edge-on	3.65<br>0.084	3.63<br>0.092	3.63<br>0.105	3.59<br>0.094
	face-on	3.61<br>0.113	3.61<br>0.106	3.59<br>0.099	3.56<br>0.103
Lamellar stacking*	edge-on	25.85<br>0.043	25.85<br>0.042	25.85<br>0.039	25.33<br>0.040
	face-on	24.25<br>0.035	24.63<br>0.032	24.25<br>0.035	24.63<br>0.030

*Inconsistency of lamella stacking distance with SAXS is possibly due to the fact that solvation has changed the lamella stacking distance.

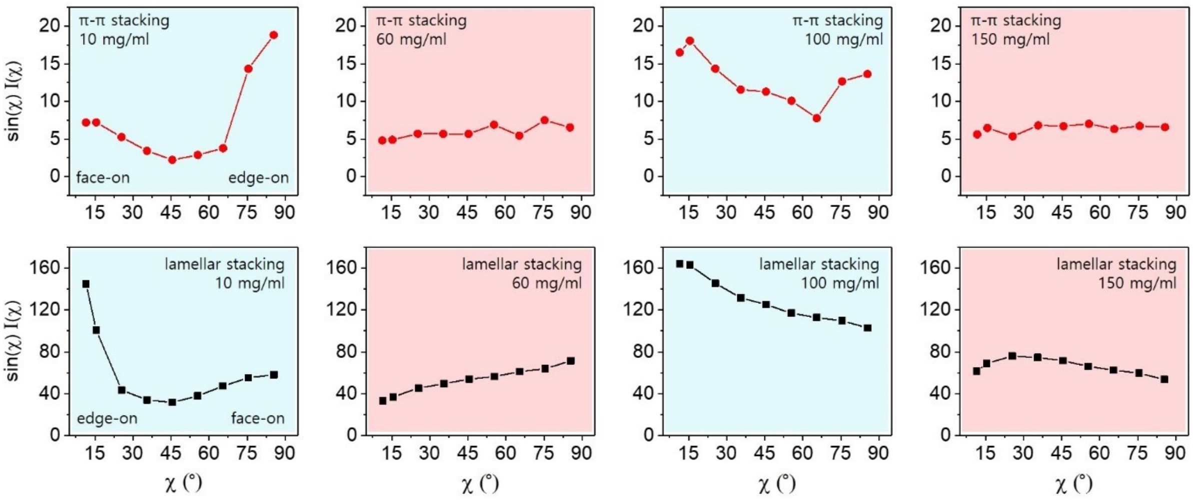

Supplementary Figure 6. Geometrically corrected intensity of π-π stacking (010) and lamellar stacking (100) peak as a function of polar angle, χ. Note that χ = 0° and 90° correspond to each face-on and edge-on orientation for π-π stacking and χ = 0° and 90° indicated each edge-on and face-on orientation for lamellar stacking.

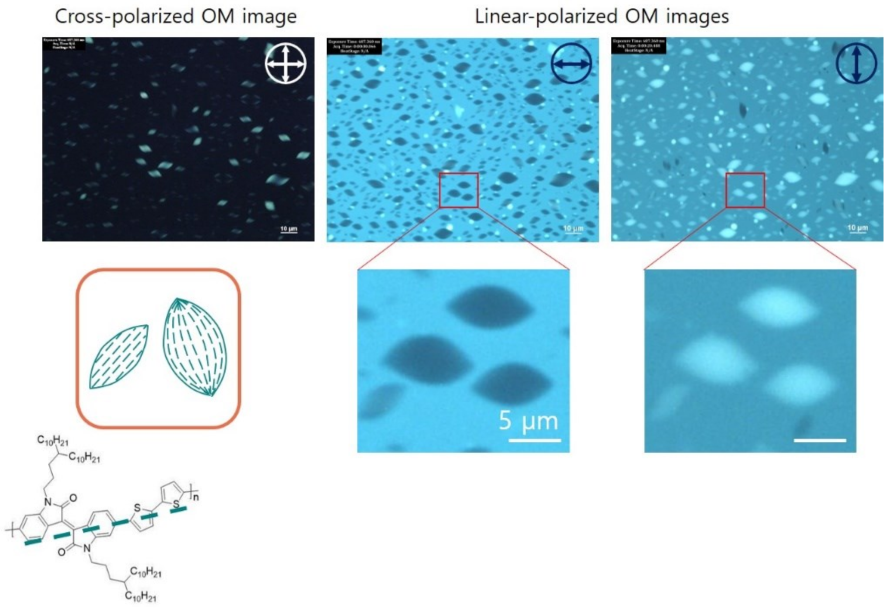

Supplementary Figure 7. Cross and linear polarized optical microscopy images of the tactoids. The tactoids show a uniform change in brightness when the tactoid is rotated from 0◦ to 90◦ relative to the polarizer. The tactoids show uniformly dark and bright when the main axis is aligned parallel and perpendicular to the polarizer, respectively. The scheme (bottom left) shows how the polymer chains are aligned inside the tactoids, indicating the chains are aligned along the fiber long axis.

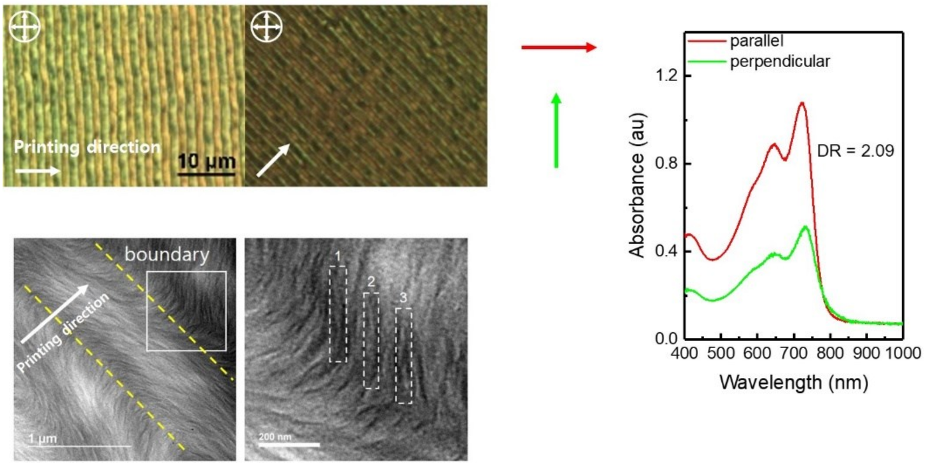

Supplementary Figure 8. CPOM, TEM and polarized UV-Vis absorption of printed PII-2T films. The nanoscale fibers are overall aligned along the printing direction despite they are wavy. Higher optical absorption when the polarizer and printing direction is parallel indicates the polymer chains are aligned along the fiber long axis.

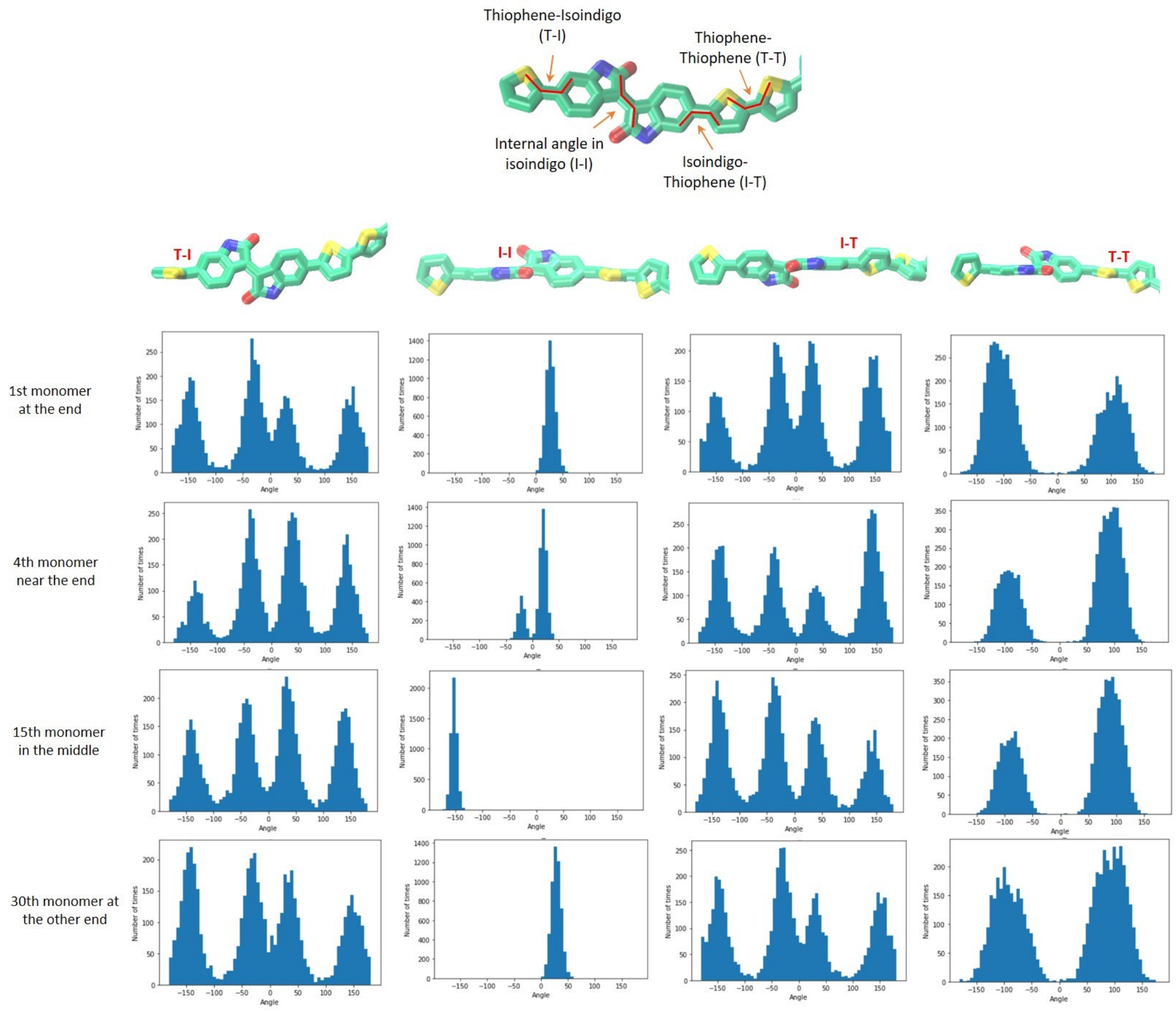

Supplementary Figure 9. Dihedral angle change at both ends, near the end and the middle region of the 30-mer PII-2T. Each angle (T-I, I-I, I-T and TT) is denoted on top of the plots with side view of molecular structures. Most of the dihedral angles are substantially changed; The I-I torsion is the most stable, centered around 25°. The T-I and I-T torsion is highly shaking, centered at two angles of +/–30° and +/–150°. The T-T torsion is also dramatically shaking, centered around +/–100°. The drastic changes of the T-I/I-T and T-T torsion might be the source of dramatic conformational change.

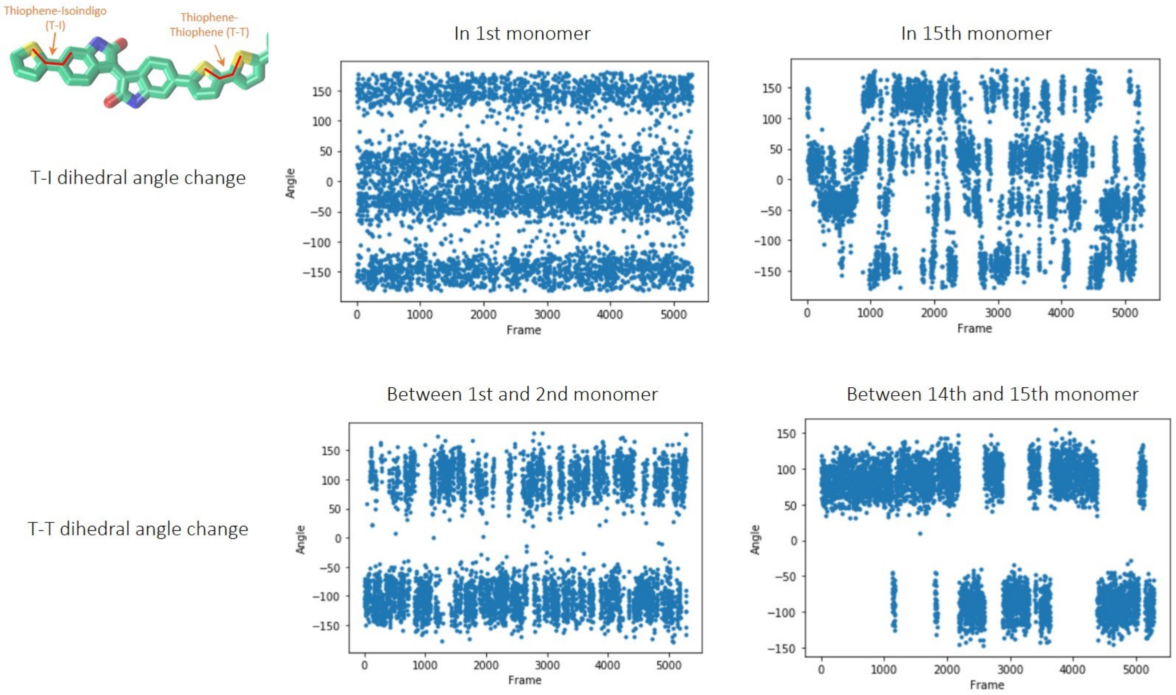

Supplementary Figure 10. The comparison of T-I and T-T dihedral angle change between the end and middle region of the 30-mer. The frequency distribution in a range of +/–150º is similar for both end and middle regions. The distribution in the end region is very much fluctuated whereas the one in the middle region is relatively stable.

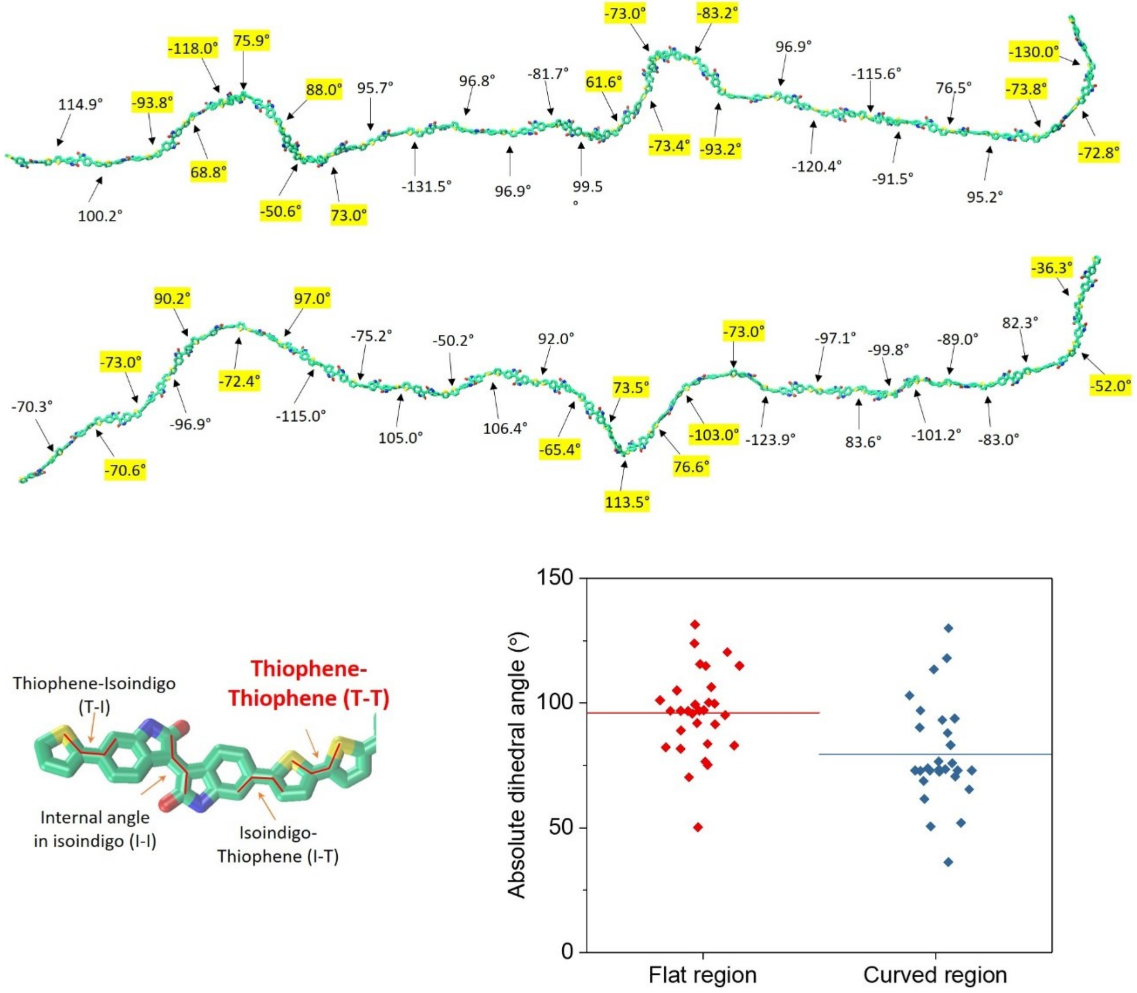

Supplementary Figure 11. Selected 30-mer chains captured from the MD simulation (top) and dihedral angle frequency plot from the flat and curved region (bottom, right). The angles were measured between the thiophene rings (T-T) of each monomer. The flat and curved region is marked by non-highlighting and highlighting, respectively. The plot shows a distribution of angle frequency, with an average value of 96.8 ± 16.9° and 79.4 ± 20.3° for the flat and curved region, respectively.

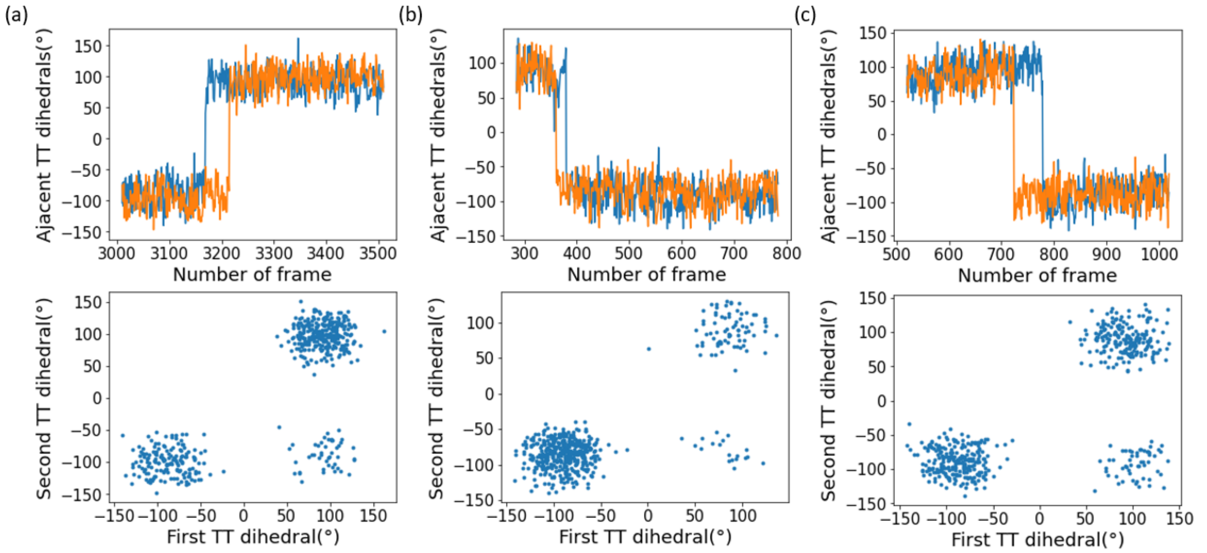

Supplementary Figure 12. (Top) Selected time-dependent dihedral angle plots of the adjacent T-T pair (yellow and blue); (left) a pair of 3rd-4th and 4th-5th position, (middle) a pair of 8th-9th and 9th-10th position and (right) a pair of 23th-24th and 24th-25th position. (Bottom) Dihedral angle distribution of each adjacent T-T pair mentioned above.

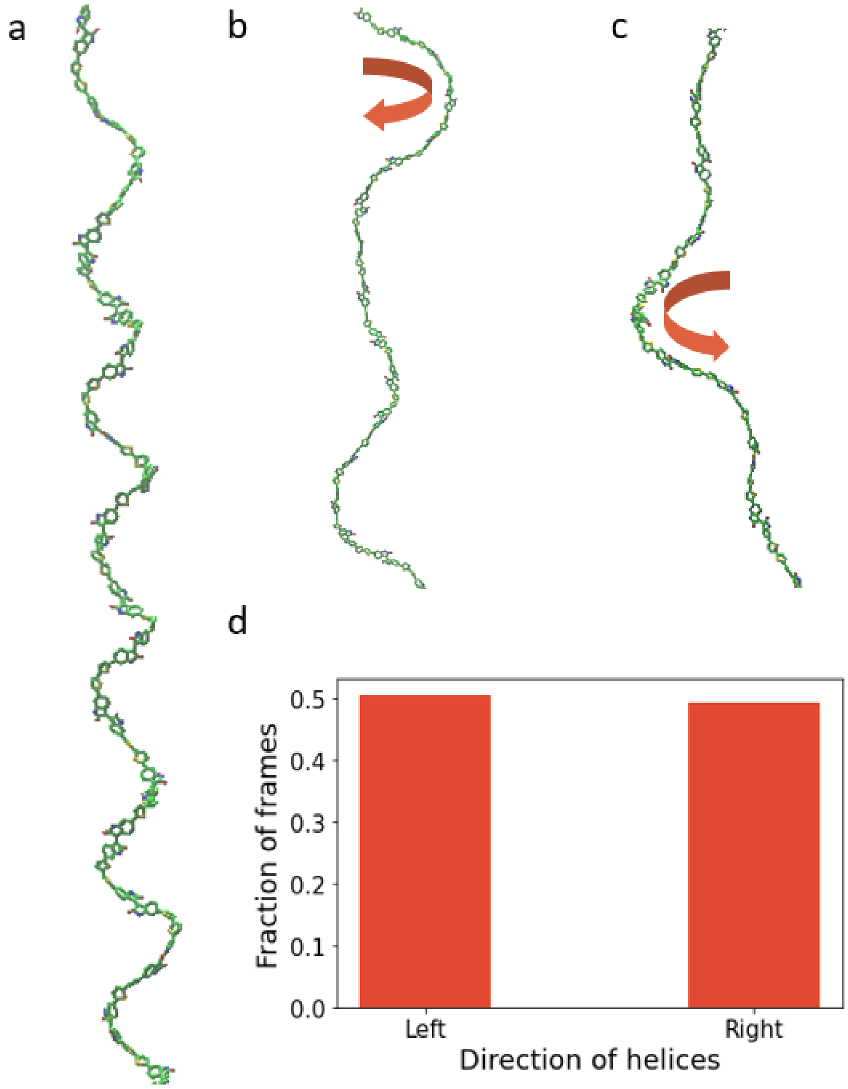

Supplementary Figure 13. (a) Backbone of PII-2T 30mer showing a right-handed helix in the relaxed structure at the start of simulation. Captured examples of the right- (b) and left-handed (c) helix formed during simulation. (d) Fraction of frames that show either left- or right-handedness in total counts of frames showing helicity.

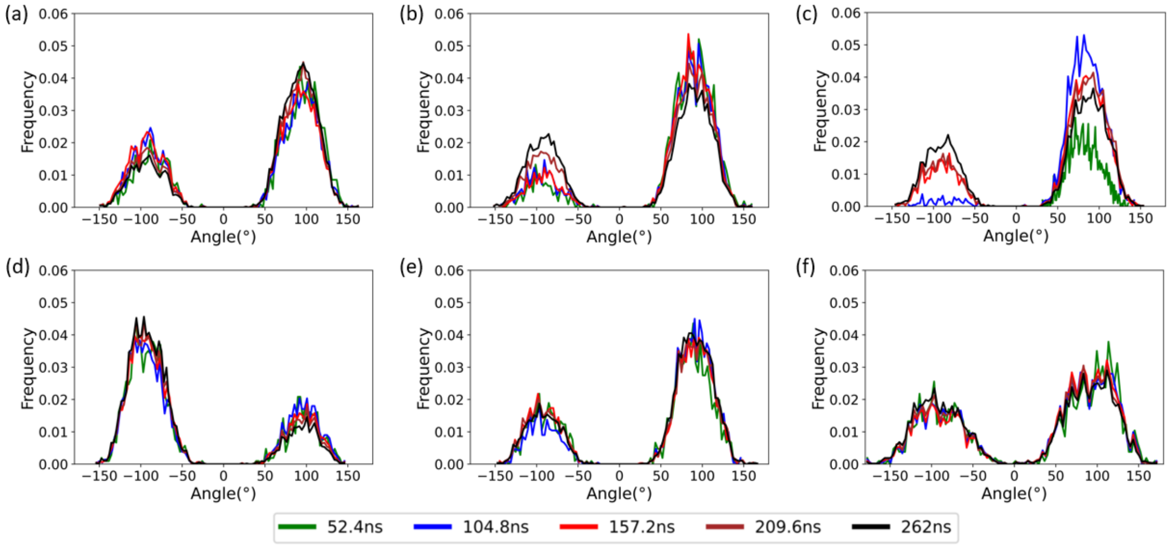

Supplementary Figure 14. Frequency distribution plots showing growth of thiophene-thiophene dihedral angles over time and the intrinsic unbalanced tendency of dihedrals. (a) T-T dihedral between 3rd and 4th monomer; (b) T-T dihedral between 9th and 10th monomer; (c) T-T dihedral between 14th and 15th monomer; (d) T-T dihedral between 19th and 20th monomer; (e) T-T dihedral between 24th and 25th monomer; (f) T-T dihedral between 29th and 30th monomer.

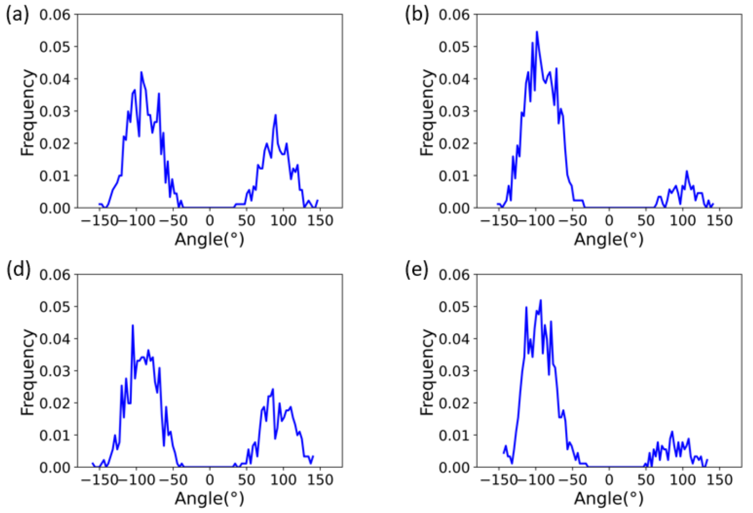

Supplementary Figure 15. Plots of T-T dihedral angles between the 14th and 15th monomer from 5 newly-simulated trajectories started from random frames picked from the first trajectory, showing the same dihedral angle asymmetry is preferred despite random starting points.

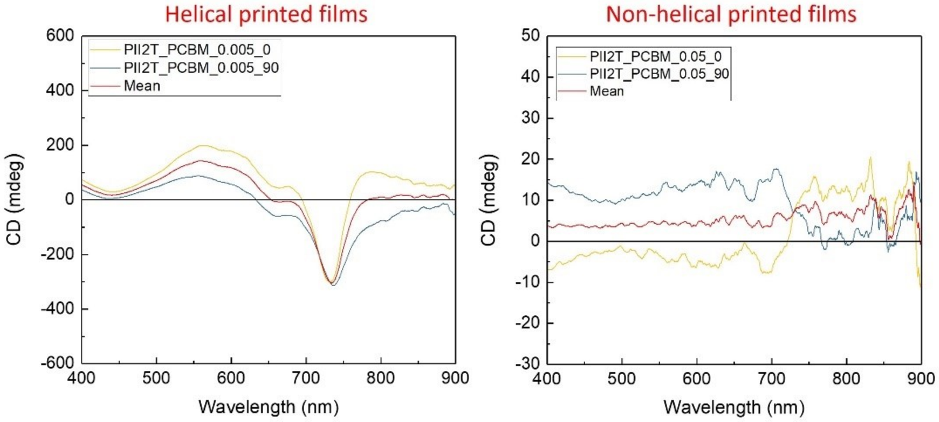

Supplementary Figure 16. CD spectra of the helical (left) and non-helical (right) PII-2T/PC71BM BHJ films, confirmed the chiral and non-chiral characteristics preserved after blending with PC71BM. CD measurements were carried out with the sample rotated in-plane 0° and 90° angles to rule out the linear dichroism and birefringence.

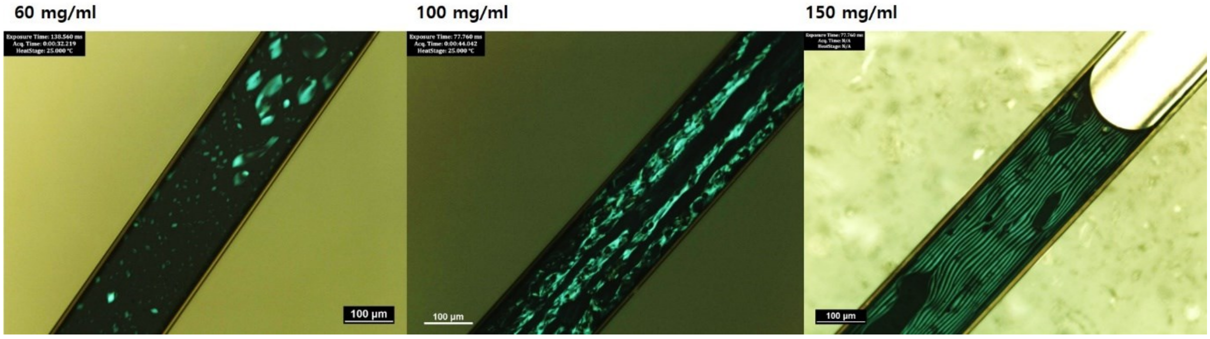

Supplementary Figure 17. CPOM images of pristine-made PII-2T solutions in 20-μm-length glass capillary.

Supplementary References

1. Ihlenfeldt, W. D., Bolton, E. E. & Bryant, S. H. The pubchem chemical structure sketcher. *J Cheminformatics* **1**, 20 (2009).
2. Hanwell, M. D., Curtis, D. E., Lonie, D. C., Vandermeersch, T., Zurek, E. & Hutchison, G. R. Avogadro: An advanced semantic chemical editor, visualization, and analysis platform. *J Cheminformatics* **4**, 17 (2012).
3. Case, D. A., Ben-Shalom, I. Y. , Brozell, S. R., Cerutti, D. S., Cheatham, T.E., III, Cruzeiro, V. W. D., Darden, T. A., Duke, R. E., Ghoreishi, D., Gilson, M. K., Gohlke, H., Goetz, A. W., Greene, D., Harris, R., Homeyer, N., Izadi, S., Kovalenko, A., Kurtzman, T., Lee, T. S., LeGrand, S., Li, P., Lin, C., Liu, J., Luchko, T., Luo, R., Mermelstein, D. J., Merz, K. M., Miao, Y., Monard, G., Nguyen, C., Nguyen, H., Omelyan, I., Onufriev, A., Pan, F., Qi, R., Roe, D. R., Roitberg, A., Sagui, C., Schott-Verdugo, S., Shen, J., Simmerling, C. L., Smith, J., Salomon-Ferrer, R., Swails, J., Walker, R. C., Wang, J., Wei, H., Wolf, R. M., Wu, X., Xiao, L., York, D. M. & Kollman, P. A. *AMBER 2018*, University of California: San Francisco, CA, 2018.
4. DeLano, W. L The pymol molecular graphics system. DeLano Scientific, San Carlos, CA, USA http://www.pymol.org (2002).
5. Martinez, L., Andrade, R., Birgin, E. G. & Martinez, J. M. Packmol: A package for building initial configurations for molecular dynamics simulations. *J Comput Chem* **30**, 2157-2164 (2009).
6. Mohammadi, E., Zhao, C. K., Meng, Y. F., Qu, G., Zhang, F. J., Zhao, X. K., Mei, J. G., Zuo, J. M., Shukla, D. & Diao, Y. Dynamic-template-directed multiscale assembly for large-area coating of highly-aligned conjugated polymer thin films. *Nat Commun* **8**, 16070 (2017).
7. Darden, T., York, D. & Pedersen, L. Particle mesh ewald - an n.Log(n) method for ewald sums in large systems. *J Chem Phys* **98**, 10089-10092 (1993).
8. Ryckaert, J. P., Ciccotti, G. & Berendsen, H. J. Numerical integration of the cartesian equations of motion of a system with constraints: Molecular dynamics of n-alkanes. *Journal of computational physics* **23**, 327-341 (1977).
9. Berendsen, H. J. C., Postma, J. P. M., Vangunsteren, W. F., Dinola, A. & Haak, J. R. Molecular-dynamics with coupling to an external bath. *J Chem Phys* **81**, 3684-3690 (1984).
10. Kumar, S., Bouzida, D., Swendsen, R. H., Kollman, P. A. & Rosenberg, J. M. The weighted histogram analysis method for free-energy calculations on biomolecules .1. The method. *J Comput Chem* **13**, 1011-1021 (1992).
11. Grossfield, A. "Wham: The weighted histogram analysis method". version 2.0.10.12, http://membrane.urmc.rochester.edu/wordpress/?page_id=126.
