# Supplementary material for: Chiral emergence in multistep hierarchical assembly of achiral conjugated polymers
Source: Nat Commun. 2022 May 18;13:2738. doi: 10.1038/s41467-022-30420-6 (PMC9117306; doi:10.1038/s41467-022-30420-6)
Supplement: Supplementary file 3 — Description of Additional Supplementary Files [file 41467_2022_30420_MOESM3_ESM.pdf]

Description of Additional Supplementary Files

Supplementary Movie 1. In-situ cross polarized optical microscopy (CPOM) video of PII-2T solution in a moving, drying meniscus. The meniscus was created by sandwiching the polymer solution between two glass slides. The video shows the solution phase (dark region on the left top) and the mesophase (bright region in the right bottom). The elliptical mesogenic domains emerge from the solution phase and coalesce to form a rope-like texture.

Supplementary Movie 2. Entire trajectory of the 30-mer simulation for 260 ns. The polymer was put in a box of chloroform. The video shows the flexible conformation of PII-2T chain in solution and the wavy-structure it takes.

Supplementary Movie 3. Entire trajectory of two short side-chain PII-2T hexamers simulated for 400ns. The two molecules were put 20 Å apart in a simulation box solvated by chloroform. This video shows the hexamers took about 100 ns to get aligned and moving together in a stable parallel conformation.
